# 6-(4-Fluoro­phen­yl)-3-phenyl-7*H*-1,2,4-tri­azolo[3,4-*b*][1,3,4]thia­diazine

**DOI:** 10.1107/S1600536814003262

**Published:** 2014-02-28

**Authors:** B. S. Palakshamurthy, H. C. Devarajegowda, N. R. Mohan, S. Sreenivasa, P. A. Suchetan

**Affiliations:** a Department of Physics, Yuvaraja’s College (Constituent College), University of Mysore Mysore, Karnataka, India; b Department of Studies and Research in Chemistry, Tumkur University, Tumkur, Karnataka 572 103, India; c Department of Studies and Research in Chemistry, U.C.S., Tumkur University, Tumkur, Karnataka 572 103, India

## Abstract

In the title compound, C_16_H_11_FN_4_S, the dihedral angles between the triazole ring and the phenyl and fluoro­benzene rings are 23.22 (17) and 18.06 (17)°, respectively. The six-membered heterocyclic ring adopts a distorted envelope conformation, with the methyl­ene C atom as the flap. In the crystal, the mol­ecules are linked by two C—H⋯N and C—H⋯F inter­actions along [010], forming *C*(5), *C*(8) and *C*(13) chains repectively. C—H⋯π inter­actions involving the phenyl ring and π–π inter­actions [centroid–centroid separation for triazole rings = 3.5660 (18) Å] are also observed.

## Related literature   

For the anti­fungal activity of nitro­gen-containing heterocylces, see: Mathew *et al.* (2007 ▶) and for their anti­bacterial activity, see: Demirbas *et al.* (2005 ▶).
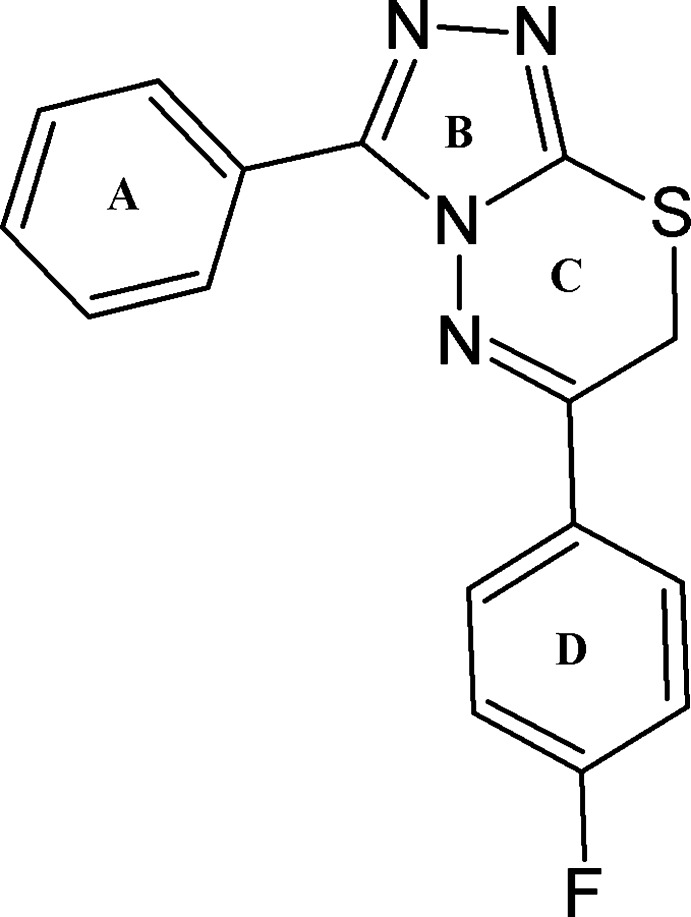


## Experimental   

### 

#### Crystal data  

C_16_H_11_FN_4_S
*M* *_r_* = 310.35
Monoclinic, 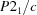

*a* = 15.088 (2) Å
*b* = 13.464 (2) Å
*c* = 7.0557 (12) Å
β = 91.076 (3)°
*V* = 1433.0 (4) Å^3^
*Z* = 4
Mo *K*α radiation
μ = 0.24 mm^−1^
*T* = 294 K
0.27 × 0.23 × 0.18 mm

#### Data collection  

Bruker APEXII CCD diffractometer
Absorption correction: multi-scan (*SADABS*; Sheldrick, 2007) ▶ *T*_min_ = 0.939, *T*_max_ = 0.958
9962 measured reflections
2154 independent reflections
1630 reflections with *I* > 2σ(*I*)
*R*_int_ = 0.060

#### Refinement  

*R*[*F* ^2^ > 2σ(*F* ^2^)] = 0.053
*wR*(*F* ^2^) = 0.134
*S* = 0.94
2154 reflections
199 parameters
H-atom parameters constrained
Δρ_max_ = 0.19 e Å^−3^
Δρ_min_ = −0.22 e Å^−3^

### 

Data collection: *APEX2* (Bruker, 2009 ▶); cell refinement: *APEX2* and *SAINT-Plus* (Bruker, 2009 ▶); data reduction: *SAINT-Plus* and *XPREP* (Bruker, 2009 ▶); program(s) used to solve structure: *SHELXS97* (Sheldrick, 2008 ▶); program(s) used to refine structure: *SHELXL97* (Sheldrick, 2008 ▶); molecular graphics: *ORTEP-3 for Windows* (Farrugia, 2012 ▶) and *Mercury* (Macrae *et al.*, 2008 ▶); software used to prepare material for publication: *SHELXL97*.

## Supplementary Material

Crystal structure: contains datablock(s) I, global. DOI: 10.1107/S1600536814003262/hb7198sup1.cif

Structure factors: contains datablock(s) I. DOI: 10.1107/S1600536814003262/hb7198Isup2.hkl

Supporting information file. DOI: 10.1107/S1600536814003262/hb7198Isup3.cml

CCDC reference:

Additional supporting information:  crystallographic information; 3D view; checkCIF report

## Figures and Tables

**Table 1:** Hydrogen-bond geometry (Å, °) *Cg*3 is the centroid of the C1–C6 phenyl ring.

*D*—H⋯*A*	*D*—H	H⋯*A*	*D*⋯*A*	*D*—H⋯*A*
C9—H9*B*⋯N2^i^	0.97	2.36	3.301 (3)	164
C12—H12⋯N2^i^	0.93	2.47	3.393 (4)	173
C4—H4⋯F1^ii^	0.93	2.57	3.475 (4)	164
C1—H1⋯*Cg*3^iii^	0.93	2.93	3.598 (3)	130

Symmetry codes: (i) 
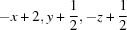
; (ii) 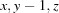; (iii) 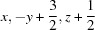.

**Fig. 1.**
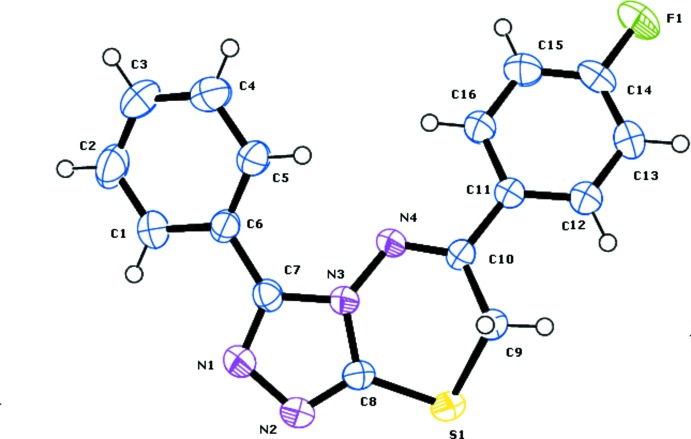
Molecular structure of the title compound, showing displacement ellipsoids drawn at the 50% probability level.

**Fig. 2.**
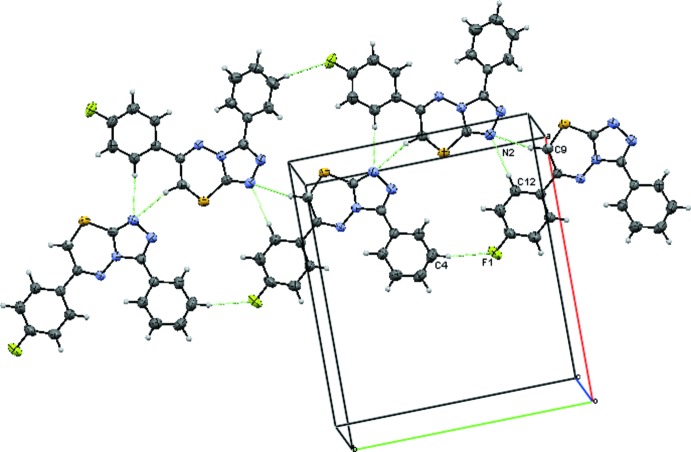
Formation of C(5), C(8) and C(13)chains through C—H···N and C—H···F hydrogen bonds.

**Fig. 3.**
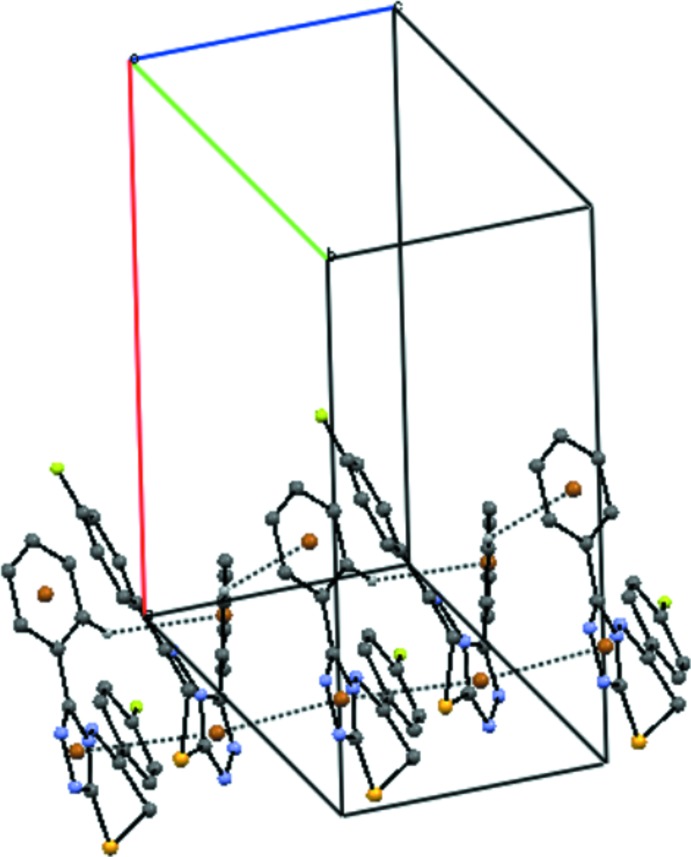
Display of C—H···π and π···π interactions in the crystal structure.

## supplementary crystallographic information

### 1. Comment

Nitrogen containing heterocyclic molecules show a broad spectrum of pharmacological properties like antifungal (Mathew *et al.*, 2007), antibacterial (Demirbas *et al.*, 2005) activities. As part of our studies in this area, the title compound was synthesized and its crystal structure determined.

In the title compound, C_14_H_11_FN_4_S, the dihedral angle between the ring pairs A—B, A—C, A—D, B—C, B—D and C—D are 23.22 (16)°, 16.62 (13)°, 29.83 (16)°, 9.86 (14)°, 18.06 (16)° and 14.61 (14)° respectively. In the crystal, the molecules are linked into one another through C9—H9···N2, C12—H12···N2 and C4—H4···F1 interactions along [010] forming C(5), C(8) and C(13) chains repectively. The structure is further stabilized by C—H···π and π···π interactions [centroid-centroid separation = 3.5660 Å] along [001] leading to a two dimensional architecture.

### 2. Experimental

An equimolar mixture of 4-amino-5-phenyl-4*H*-1,2,4-triazole-3-thiol (1 mmole) and 2-chloro-1-(4-fluorophenyl)ethanone (1 mmole) and sodium acetate (2.5 mmol) in absolute ethanol (10 ml) were refluxed for 2 h and completion of the reaction was monitored by TLC. Reaction mixture was cooled to room temperature, the solvent was removed under vacuum and the precipitate obtained was filtered, washed with water and dried to get crude product. The Crude solid was further purified by column chromatography using dichloromethane/methanol (9:1) as eluent and was later recrystallized from dichloromethane/methanol solvent system to get colorless prisms of the title compound.

### 3. Refinement

The other H atoms were positioned with idealized geometry using a riding model with C—H = 0.93–0.96 Å. All H atoms were refined with isotropic displacement parameters (set to 1.2–1.5 times of the U eq of the parent atom).

### Crystal data

**Table:**

C_16_H_11_FN_4_S	Prism
*M**_r_* = 310.35	*D*_x_ = 1.438 Mg m^−^^3^
Monoclinic, *P*2_1_/*c*	Melting point: 523 K
Hall symbol: -P 2ybc	Mo *K*α radiation, λ = 0.71073 Å
*a* = 15.088 (2) Å	Cell parameters from 199 reflections
*b* = 13.464 (2) Å	θ = 1.2–26°
*c* = 7.0557 (12) Å	µ = 0.24 mm^−^^1^
β = 91.076 (3)°	*T* = 294 K
*V* = 1433.0 (4) Å^3^	Prism, colourless
*Z* = 4	0.27 × 0.23 × 0.18 mm
*F*(000) = 640	

### Data collection

**Table:**

Bruker APEXII CCD diffractometer	2154 independent reflections
Radiation source: fine-focus sealed tube	1630 reflections with *I* > 2σ(*I*)
Graphite monochromator	*R*_int_ = 0.060
Detector resolution: 1.6 pixels mm^-1^	θ_max_ = 26.0°, θ_min_ = 2.0°
phi and ω scans	*h* = −18→18
Absorption correction: multi-scan (*SADABS*; Sheldrick, 2007)	*k* = −16→16
*T*_min_ = 0.939, *T*_max_ = 0.958	*l* = −8→8
9962 measured reflections	

### Refinement

**Table:**

Refinement on *F*^2^	Primary atom site location: structure-invariant direct methods
Least-squares matrix: full	Secondary atom site location: difference Fourier map
*R*[*F*^2^ > 2σ(*F*^2^)] = 0.053	Hydrogen site location: inferred from neighbouring sites
*wR*(*F*^2^) = 0.134	H-atom parameters constrained
*S* = 0.94	*w* = 1/[σ^2^(*F*_o_^2^) + (0.0688*P*)^2^] where *P* = (*F*_o_^2^ + 2*F*_c_^2^)/3
2154 reflections	(Δ/σ)_max_ < 0.001
199 parameters	Δρ_max_ = 0.19 e Å^−^^3^
0 restraints	Δρ_min_ = −0.22 e Å^−^^3^
11 constraints	

### Special details

**Table:**

Geometry. All e.s.d.'s (except the e.s.d. in the dihedral angle between two l.s. planes) are estimated using the full covariance matrix. The cell e.s.d.'s are taken into account individually in the estimation of e.s.d.'s in distances, angles and torsion angles; correlations between e.s.d.'s in cell parameters are only used when they are defined by crystal symmetry. An approximate (isotropic) treatment of cell e.s.d.'s is used for estimating e.s.d.'s involving l.s. planes.
Refinement. Refinement of *F*^2^ against ALL reflections. The weighted *R*-factor *wR* and goodness of fit *S* are based on *F*^2^, conventional *R*-factors *R* are based on *F*, with *F* set to zero for negative *F*^2^. The threshold expression of *F*^2^ > σ(*F*^2^) is used only for calculating *R*-factors(gt) *etc*. and is not relevant to the choice of reflections for refinement. *R*-factors based on *F*^2^ are statistically about twice as large as those based on *F*, and *R*- factors based on ALL data will be even larger.

### Fractional atomic coordinates and isotropic or equivalent isotropic displacement parameters (Å^2^)

**Table:**

	*x*	*y*	*z*	*U*_iso_*/*U*_eq_	
S1	1.01380 (5)	0.90521 (5)	0.19233 (12)	0.0434 (2)	
N3	0.86107 (14)	0.80121 (14)	0.1963 (3)	0.0328 (5)	
N2	0.97880 (15)	0.70583 (16)	0.2103 (4)	0.0444 (6)	
N1	0.90331 (15)	0.64596 (17)	0.2066 (4)	0.0416 (6)	
N4	0.80738 (14)	0.88156 (15)	0.1519 (3)	0.0330 (5)	
C10	0.83938 (16)	0.96827 (18)	0.1890 (4)	0.0321 (6)	
C7	0.83399 (17)	0.70319 (18)	0.1968 (4)	0.0340 (6)	
C6	0.74169 (17)	0.66820 (19)	0.1859 (4)	0.0351 (6)	
C9	0.92724 (16)	0.9844 (2)	0.2857 (4)	0.0391 (6)	
H9A	0.9215	0.9715	0.4202	0.047*	
H9B	0.9444	1.0533	0.2709	0.047*	
C11	0.78112 (17)	1.05326 (18)	0.1437 (4)	0.0343 (6)	
C8	0.95214 (16)	0.79732 (19)	0.2038 (4)	0.0357 (6)	
F1	0.61197 (13)	1.28437 (13)	0.0296 (4)	0.0784 (7)	
C5	0.7266 (2)	0.5726 (2)	0.1183 (4)	0.0450 (7)	
H5	0.7739	0.5326	0.0841	0.054*	
C15	0.6392 (2)	1.1137 (2)	0.0357 (5)	0.0543 (8)	
H15	0.5822	1.1019	−0.0114	0.065*	
C1	0.67026 (18)	0.7263 (2)	0.2400 (5)	0.0432 (7)	
H1	0.6800	0.7897	0.2881	0.052*	
C16	0.69570 (18)	1.0365 (2)	0.0751 (5)	0.0458 (7)	
H16	0.6764	0.9716	0.0556	0.055*	
C2	0.5845 (2)	0.6898 (2)	0.2223 (6)	0.0571 (9)	
H2	0.5366	0.7289	0.2570	0.068*	
C12	0.80794 (19)	1.1509 (2)	0.1700 (5)	0.0473 (7)	
H12	0.8653	1.1640	0.2139	0.057*	
C3	0.5708 (2)	0.5959 (2)	0.1535 (6)	0.0597 (9)	
H3	0.5133	0.5714	0.1412	0.072*	
C14	0.6683 (2)	1.2082 (2)	0.0669 (5)	0.0510 (8)	
C13	0.7514 (2)	1.2285 (2)	0.1325 (6)	0.0559 (9)	
H13	0.7697	1.2938	0.1517	0.067*	
C4	0.6409 (2)	0.5377 (2)	0.1024 (5)	0.0583 (9)	
H4	0.6306	0.4739	0.0566	0.070*	

### Atomic displacement parameters (Å^2^)

**Table:**

	*U*^11^	*U*^22^	*U*^33^	*U*^12^	*U*^13^	*U*^23^
S1	0.0270 (3)	0.0420 (4)	0.0614 (5)	−0.0019 (3)	0.0009 (3)	0.0042 (3)
N3	0.0284 (11)	0.0298 (11)	0.0401 (12)	0.0015 (9)	−0.0039 (9)	−0.0011 (9)
N2	0.0332 (12)	0.0383 (13)	0.0616 (16)	0.0045 (10)	−0.0029 (11)	0.0027 (11)
N1	0.0375 (12)	0.0335 (12)	0.0535 (14)	0.0038 (10)	−0.0051 (11)	0.0004 (11)
N4	0.0289 (11)	0.0295 (11)	0.0406 (12)	0.0041 (9)	−0.0034 (9)	−0.0001 (9)
C10	0.0302 (12)	0.0313 (13)	0.0349 (13)	−0.0028 (10)	0.0005 (11)	−0.0021 (11)
C7	0.0375 (14)	0.0303 (13)	0.0340 (13)	0.0012 (11)	−0.0024 (11)	−0.0027 (10)
C6	0.0353 (13)	0.0323 (13)	0.0376 (14)	−0.0041 (11)	−0.0029 (11)	0.0018 (11)
C9	0.0326 (13)	0.0348 (14)	0.0494 (16)	−0.0012 (11)	−0.0055 (12)	−0.0004 (12)
C11	0.0322 (13)	0.0318 (13)	0.0387 (14)	0.0002 (11)	−0.0001 (11)	−0.0007 (11)
C8	0.0286 (13)	0.0366 (14)	0.0418 (15)	0.0009 (11)	−0.0015 (11)	0.0019 (11)
F1	0.0629 (12)	0.0484 (11)	0.123 (2)	0.0259 (9)	−0.0115 (13)	0.0061 (12)
C5	0.0494 (17)	0.0335 (14)	0.0520 (18)	−0.0018 (13)	0.0006 (14)	−0.0031 (13)
C15	0.0394 (16)	0.0508 (18)	0.072 (2)	0.0069 (14)	−0.0123 (16)	−0.0005 (16)
C1	0.0391 (14)	0.0401 (15)	0.0504 (17)	−0.0038 (12)	−0.0037 (13)	−0.0075 (13)
C16	0.0376 (15)	0.0344 (14)	0.065 (2)	0.0021 (12)	−0.0100 (15)	−0.0013 (13)
C2	0.0392 (16)	0.059 (2)	0.073 (2)	−0.0003 (15)	−0.0022 (16)	−0.0074 (17)
C12	0.0388 (15)	0.0372 (15)	0.0656 (19)	0.0006 (13)	−0.0069 (14)	−0.0013 (15)
C3	0.0426 (17)	0.061 (2)	0.075 (2)	−0.0164 (15)	−0.0093 (17)	−0.0050 (18)
C14	0.0467 (16)	0.0428 (16)	0.064 (2)	0.0178 (14)	0.0001 (15)	0.0044 (15)
C13	0.0521 (17)	0.0326 (15)	0.083 (2)	0.0020 (13)	−0.0015 (18)	−0.0027 (15)
C4	0.061 (2)	0.0440 (17)	0.070 (2)	−0.0170 (16)	−0.0060 (18)	−0.0085 (16)

### Geometric parameters (Å, º)

**Table:**

S1—C8	1.728 (3)	F1—C14	1.354 (3)
S1—C9	1.819 (3)	C5—C4	1.378 (4)
N3—C8	1.375 (3)	C5—H5	0.9300
N3—C7	1.382 (3)	C15—C14	1.362 (4)
N3—N4	1.384 (3)	C15—C16	1.370 (4)
N2—C8	1.296 (3)	C15—H15	0.9300
N2—N1	1.395 (3)	C1—C2	1.388 (4)
N1—C7	1.300 (3)	C1—H1	0.9300
N4—C10	1.288 (3)	C16—H16	0.9300
C10—C11	1.474 (3)	C2—C3	1.369 (4)
C10—C9	1.495 (3)	C2—H2	0.9300
C7—C6	1.471 (4)	C12—C13	1.371 (4)
C6—C1	1.391 (4)	C12—H12	0.9300
C6—C5	1.390 (4)	C3—C4	1.371 (5)
C9—H9A	0.9700	C3—H3	0.9300
C9—H9B	0.9700	C14—C13	1.356 (5)
C11—C16	1.387 (4)	C13—H13	0.9300
C11—C12	1.387 (4)	C4—H4	0.9300
			
C8—S1—C9	94.87 (13)	C4—C5—H5	120.3
C8—N3—C7	105.0 (2)	C6—C5—H5	120.3
C8—N3—N4	128.2 (2)	C14—C15—C16	118.6 (3)
C7—N3—N4	125.1 (2)	C14—C15—H15	120.7
C8—N2—N1	107.2 (2)	C16—C15—H15	120.7
C7—N1—N2	108.3 (2)	C6—C1—C2	120.2 (3)
C10—N4—N3	116.5 (2)	C6—C1—H1	119.9
N4—C10—C11	116.1 (2)	C2—C1—H1	119.9
N4—C10—C9	123.4 (2)	C15—C16—C11	121.2 (3)
C11—C10—C9	120.4 (2)	C15—C16—H16	119.4
N1—C7—N3	109.2 (2)	C11—C16—H16	119.4
N1—C7—C6	125.0 (2)	C3—C2—C1	119.6 (3)
N3—C7—C6	125.8 (2)	C3—C2—H2	120.2
C1—C6—C5	119.5 (3)	C1—C2—H2	120.2
C1—C6—C7	122.9 (2)	C13—C12—C11	121.1 (3)
C5—C6—C7	117.6 (2)	C13—C12—H12	119.4
C10—C9—S1	112.70 (19)	C11—C12—H12	119.4
C10—C9—H9A	109.1	C2—C3—C4	120.6 (3)
S1—C9—H9A	109.1	C2—C3—H3	119.7
C10—C9—H9B	109.1	C4—C3—H3	119.7
S1—C9—H9B	109.1	F1—C14—C13	119.1 (3)
H9A—C9—H9B	107.8	F1—C14—C15	118.5 (3)
C16—C11—C12	117.9 (3)	C13—C14—C15	122.4 (3)
C16—C11—C10	119.7 (2)	C14—C13—C12	118.7 (3)
C12—C11—C10	122.4 (2)	C14—C13—H13	120.6
N2—C8—N3	110.3 (2)	C12—C13—H13	120.6
N2—C8—S1	129.3 (2)	C3—C4—C5	120.7 (3)
N3—C8—S1	120.31 (19)	C3—C4—H4	119.6
C4—C5—C6	119.4 (3)	C5—C4—H4	119.6
			
C8—N2—N1—C7	0.4 (3)	C7—N3—C8—N2	−0.6 (3)
C8—N3—N4—C10	−28.0 (4)	N4—N3—C8—N2	−166.4 (3)
C7—N3—N4—C10	168.8 (2)	C7—N3—C8—S1	176.35 (19)
N3—N4—C10—C11	−179.2 (2)	N4—N3—C8—S1	10.5 (4)
N3—N4—C10—C9	−3.7 (4)	C9—S1—C8—N2	−158.0 (3)
N2—N1—C7—N3	−0.8 (3)	C9—S1—C8—N3	25.7 (2)
N2—N1—C7—C6	178.6 (3)	C1—C6—C5—C4	1.3 (5)
C8—N3—C7—N1	0.9 (3)	C7—C6—C5—C4	−178.8 (3)
N4—N3—C7—N1	167.3 (2)	C5—C6—C1—C2	−1.5 (5)
C8—N3—C7—C6	−178.5 (3)	C7—C6—C1—C2	178.6 (3)
N4—N3—C7—C6	−12.1 (4)	C14—C15—C16—C11	−0.6 (5)
N1—C7—C6—C1	157.2 (3)	C12—C11—C16—C15	−0.4 (5)
N3—C7—C6—C1	−23.5 (4)	C10—C11—C16—C15	178.5 (3)
N1—C7—C6—C5	−22.7 (4)	C6—C1—C2—C3	0.8 (5)
N3—C7—C6—C5	156.5 (3)	C16—C11—C12—C13	1.1 (5)
N4—C10—C9—S1	44.7 (3)	C10—C11—C12—C13	−177.9 (3)
C11—C10—C9—S1	−140.1 (2)	C1—C2—C3—C4	0.2 (6)
C8—S1—C9—C10	−48.7 (2)	C16—C15—C14—F1	−179.3 (3)
N4—C10—C11—C16	3.3 (4)	C16—C15—C14—C13	1.1 (6)
C9—C10—C11—C16	−172.2 (3)	F1—C14—C13—C12	180.0 (3)
N4—C10—C11—C12	−177.8 (3)	C15—C14—C13—C12	−0.5 (6)
C9—C10—C11—C12	6.7 (4)	C11—C12—C13—C14	−0.6 (5)
N1—N2—C8—N3	0.1 (3)	C2—C3—C4—C5	−0.4 (6)
N1—N2—C8—S1	−176.5 (2)	C6—C5—C4—C3	−0.4 (5)

### Hydrogen-bond geometry (Å, º)

Cg3 is the centroid of the C1–C6 phenyl ring.

**Table:**

*D*—H···*A*	*D*—H	H···*A*	*D*···*A*	*D*—H···*A*
C9—H9*B*···N2^i^	0.97	2.36	3.301 (3)	164
C12—H12···N2^i^	0.93	2.47	3.393 (4)	173
C4—H4···F1^ii^	0.93	2.57	3.475 (4)	164
C1—H1···*Cg*3^iii^	0.93	2.93	3.598 (3)	130

Symmetry codes: (i) −*x*+2, *y*+1/2, −*z*+1/2; (ii) *x*, *y*−1, *z*; (iii) *x*, −*y*+3/2, *z*+1/2.
